# Takotsubo Syndrome: From Bench to Bedside and Bedside to Bench

**DOI:** 10.3390/jcm11164704

**Published:** 2022-08-11

**Authors:** Francesco Pelliccia, Amalia Morgantini, Riccardo Rosati

**Affiliations:** Department of Cardiovascular Sciences, Sapienza University, 00163 Rome, Italy

Takotsubo syndrome (TTS) typically manifests as acute chest pain and/or dyspnea triggered by intense psychological or physiological stress. Although symptoms mimic an acute myocardial infarction (MI), no obstructive lesions are found in the coronary arteries, and left ventricular (LV) apical ballooning is usually present [1]. The current understanding of the etiology of this intriguing condition suggests that sympathetic nervous system (SNS) activation plays a central role. Nevertheless, controversy still exists around multiple aspects of the condition, including its definition, pathophysiology, triggers, diagnostic work-up, treatment and outcome. Recently, a large body of studies have shed light on the several unresolved issues of the condition.

According to the current European Society of Cardiology guidelines, TTS is no longer included within the spectrum of patients classified as MI< with non-obstructive coronary arteries (MINOCA). As a consequence, our understanding of the epidemiology of the two conditions needs to be updated. In this respect, Dees et al. provided novel information on the incidence of TTS and MINOCA [2]. They reviewed data from 1532 patients with MI and found 117 patients with no significant coronary obstructions. Of them, 88 patients had type II MI (75%), 23 patients were diagnosed with MINOCA (20%), and 6 patients had TTS (5%).

As regards the diagnosis of TTS, a novel algorithm might help physicians to distinguish the condition from an acute coronary syndrome (ACS) at time of triage. Topf et al. reported data from 93 patients with chest pain and the suspicion of TTS who had assessment of serum levels of novel biomarkers (i.e., Fetuin-A, IGFBP-2, Galectin-3, and TNF) within 24 h after the onset of symptoms [3]. Compared to TTS, patients with ACS had significantly lower serum levels of Fetuin-A and IGFBP-2, thus indicating that these biomarkers might facilitate the triage between TTS and ACS and therefore might be of great benefit for the guidance of treatment. A proper diagnosis of TTS can also be facilitated by the analysis of life event characteristics. Casagrande et al. compared 54 patients with TTS with 52 patients with MI and 54 healthy individuals [4]. Using a modified version of the Interview for Recent Life Events, information about general life events perceived as stressful and triggers preceding the onset of a cardiac syndrome was collected. Interestingly, 61% of TTS patients objectively and subjectively reported a more stressful trigger before the onset of the disease (in the 24 h preceding the cardiac event) than those reported by patients with MI. These findings suggest that patients’ perception of some life events (whether triggers or general life events) could represent a possible marker of TTS.

Beyond clinical presentation, epidemiology and novel diagnostic biomarkers, Rawish et al. have recently provided a comprehensive review that draws attention to potential pathophysiological mechanisms for the observed reversible myocardial dysfunction such as sympathetic overdrive-mediated multi-vessel epicardial spasms, microvascular dysfunction, the direct toxicity of catecholamines, lipotoxicity and inflammation [5]. In this respect, the results of the prospective CIRCUS-TTS study are of major importance. Mölle et al. compared the systemic microvascular function of 22 TTS patients, 20 patients with MI and 20 healthy subjects by means of the sublingual side stream dark-field imaging [6]. Surprisingly, the results did not show relevant differences between the three groups in the assessed parameters, i.e., the number of vessel crossings, the number of perfused vessel crossings, the proportion of perfused vessels, the total vessel density and the perfused vessel density.

At variance with these findings, the study by Sans-Roselló et al. showed that coronary microvascular disease (CMD) is highly prevalent in patients admitted for TTS and is associated with both a higher degree of LV systolic dysfunction [7]. Of 181 consecutive patients admitted for TTS, the authors found that all patients presented CMD in at least one epicardial coronary artery, with the left anterior descending artery showing higher median index of microvascular resistance (IMR) values than left circumflex and right coronary arteries. Another study has suggested that myocardial ischemia might have a central role in developing TTS. Kato et al. investigated the recovery process of TTS in 15 patients who underwent serial cardiovascular magnetic resonance (CMR) [8]. CMR demonstrated a reduced LV ejection fraction in the acute phase, which recovered almost completely by the subacute phase. On the other hand, severe myocardial edema was still observed in the subacute phase, despite the functional recovery of the LV function.

Similarly to microcirculation, there is a growing body of evidence that inflammation might play a role in the development of TTS. Pergola et al. evaluated the presence and extent of coronary inflammation in a court of patients with myocardial damage and no angiographically significant coronary atherosclerosis [9]. These authors assessed the perivascular fat attenuation index (pFAI), i.e., a new imaging marker of inflammation, in patients diagnosed with myocarditis, MINOCA, TTS and healthy subjects. The pFAI values were significantly lower in healthy controls compared to patients with myocarditis, MINOCA and TTS and were abnormal for a longer time in TTS as compared with the other conditions.

As regards clinical outcome of TTS, recent evidences have shown that the rates of acute complications and in-hospital mortality are comparable to that of ACS patients. In particular, the prevalence of cardiogenic shock ranges between 6% and 20%. In this setting, the detection of mechanisms leading to cardiogenic shock can be challenging. Recently, Di Vece et al. pointed out that the onset of LV outflow tract obstruction together with mitral regurgitation related to systolic anterior motion of mitral valve leaflets can lead to hemodynamic instability, in addition to a severely impaired LV systolic function [10]. Moreover, patients with TTS can even experience cardiac rupture, which is no doubt the most serious complication of an ACS [11].

These new evidences highlight the crucial role of a proper management of TTS during the acute phase, which is mainly performed with supportive pharmacological (diuretics, ACE-inhibitors/angiotensin-receptor blockers, anticoagulants, antiarrhythmics, non-catecholamine inotropics (levosimendan), and non-pharmacological (mechanical circulatory and respiratory support) therapy. As stated by Santoro et al., however, there is a gap in evidence and there are no randomized and adequately powered studies on clinical effectiveness of therapeutic approaches [12]. Some evidence supports the use of ACE-inhibitors/angiotensin-receptor blockers at long-term in selected cases, but a tailored approach based on cardiovascular and non-cardiovascular risk factors is mandatory for long-term management. In addition, some new therapeutic hypotheses (i.e., large doses of insulin infusions in association with intravenous short- and ultrashort-acting beta-blockers) are being proposed based on previous extensive animal work and limited application in patients with neurogenic cardiomyopathy and TTS [13]. These findings highlight the fact that an optimal medical treatment of TTS is of particular concern when patients suffer from comorbidities such as malignancy and are therefore at higher risk of readmission. Jang et al. examined the rates, causes, and costs of 30-day readmissions of TTS, with or without malignancy, by utilizing Nationwide Readmissions Databases from 2010 to 2014 [14]. The authors identified 61,588 patients with TTS and showed that those with malignancy tended to be older and with a higher overall burden of comorbidities than those without malignancy. Noteworthy, TTS patients with malignancy had significantly higher 30-day readmission rates (15.9% vs. 11.0%) and total costs (by 25%) than those without malignancy.

In conclusion, newly available diagnostic tools have improved our knowledge of the pathophysiology of TTS. The findings of most recent studies conducted in TTS, from bench to bedside and bedside to bench, have the potential to aid clinicians in the decision-making process, as well as future directions for research given the current lack of evidence-based medical approaches [15].
